# Functional assays for the assessment of the pathogenicity of variants of GOSR2, an ER-to-Golgi SNARE involved in progressive myoclonus epilepsies

**DOI:** 10.1242/dmm.029132

**Published:** 2017-12-01

**Authors:** Jörn M. Völker, Mykola Dergai, Luciano A. Abriata, Yves Mingard, Daniel Ysselstein, Dimitri Krainc, Matteo Dal Peraro, Gabriele Fischer von Mollard, Dirk Fasshauer, Judith Koliwer, Michael Schwake

**Affiliations:** 1 Biochemistry III/Faculty of Chemistry, Bielefeld University, Universitätsstraße 25, 33615 Bielefeld, Germany; 2 Department of Fundamental Neurosciences, University of Lausanne, Rue du Bugnon 9, 1005 Lausanne, Switzerland; 3 Institute of Bioengineering, School of Life Sciences, École Polytechnique Fédérale de Lausanne (EPFL), CH-1015 Lausanne, Switzerland; 4 Swiss Institute of Bioinformatics (SIB), CH-1015 Lausanne, Switzerland; 5 Department of Neurology, Northwestern University Feinberg School of Medicine, 303 East Chicago Avenue, 60611 Chicago, USA

**Keywords:** Progressive myoclonus epilepsies, PME, GOSR2, Membrin, GS27, Bos1

## Abstract

Progressive myoclonus epilepsies (PMEs) are inherited disorders characterized by myoclonus, generalized tonic-clonic seizures, and ataxia. One of the genes that is associated with PME is the ER-to-Golgi Q_b_-SNARE GOSR2, which forms a SNARE complex with syntaxin-5, Bet1 and Sec22b. Most PME patients are homo­zygous for a p.Gly144Trp mutation and develop similar clinical presentations. Recently, a patient who was compound heterozygous for p.Gly144Trp and a previously unseen p.Lys164del mutation was identified. Because this patient presented with a milder disease phenotype, we hypothesized that the p.Lys164del mutation may be less severe compared to p.Gly144Trp. To characterize the effect of the p.Gly144Trp and p.Lys164del mutations, both of which are present in the SNARE motif of GOSR2, we examined the corresponding mutations in the yeast ortholog Bos1. Yeasts expressing the orthologous mutants in Bos1 showed impaired growth, suggesting a partial loss of function, which was more severe for the Bos1 p.Gly176Trp mutation. Using anisotropy and gel filtration, we report that Bos1 p.Gly176Trp and p.Arg196del are capable of complex formation, but with partly reduced activity. Molecular dynamics (MD) simulations showed that the hydrophobic core, which triggers SNARE complex formation, is compromised due to the glycine-to-tryptophan substitution in both GOSR2 and Bos1. In contrast, the deletion of residue p.Lys164 (or p.Arg196del in Bos1) interferes with the formation of hydrogen bonds between GOSR2 and syntaxin-5. Despite these perturbations, all SNARE complexes stayed intact during longer simulations. Thus, our data suggest that the milder course of disease in compound heterozygous PME is due to less severe impairment of the SNARE function.

## Abstract (precis)

**Summary:** Mutations in the Q_b_-SNARE GOSR2 cause progressive myoclonus epilepsies. The authors report the effect of two mutations on SNARE function to investigate their correlation with progression and severity of disease.

## INTRODUCTION

**Fig. 1.**
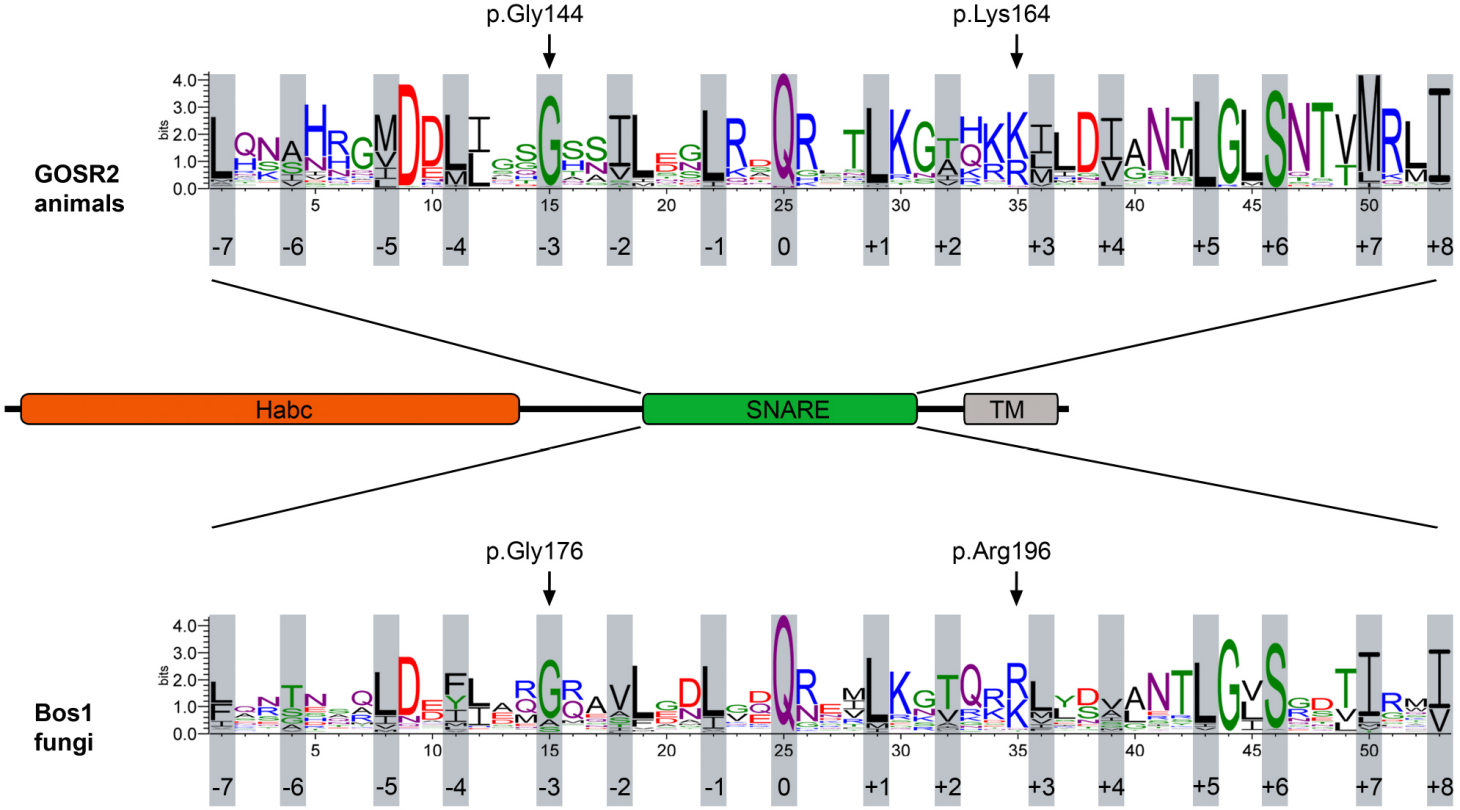
**SNARE motif sequence alignment of GOSR2 and its yeast ortholog, Bos1.** The PME-associated mutations of GOSR2, p.Gly144 and p.Lys164, as well as the yeast ortholog Bos1 p.Gly176 and p.Arg196, are located at highly conserved positions within the SNARE motif. GOSR2 and Bos1 contain an N-terminal Habc domain, a SNARE motif and a transmembrane domain (TM) at the C-terminus. Sequence alignments of SNARE proteins of over 200 animals or fungi are presented as weblogo and show the high sequence conservation of the SNARE domain (Crooks et al., 2004). GOSR2 and Bos1 share high sequence similarity. The SNARE motif consists of 16 hydrophobic layers, as indicated. The p.Gly144 and p.Gly176 mutations are located at the −3 layer, whereas p.Lys164 and p.Arg196 are located between layers +2 and +3 (Kloepper et al., 2007).

Subcellular trafficking of membranes and their associated proteins is essential for proper function of eukaryotic cells. Fission from donor membranes and fusion of transport vesicles with target membranes allows for controlled transport of cargo, including lipids, proteins, and cellular messengers such as hormones. SNARE [soluble NSF (N-ethylmaleimide-sensitive factor) attachment protein receptor] proteins, with their highly conserved SNARE domains, are a main component of the fusion process. Four different SNARE domains found on vesicles and target membranes interact to form a quaternary SNARE complex providing the driving force necessary for membrane fusion (Jahn and Scheller, 2006). The SNARE complex structure is defined by a twisted parallel bundle of four helices (Sutton et al., 1998). The contacting surfaces of these helices can be separated into 16 layers, which are indicated by numbers from −7 to +8 (Fig. 1). These layers are mainly hydrophobic, except for the hydrophilic 0-layer in the center of the bundle (Fasshauer et al., 1998). The complex usually consists of three Q-SNAREs (Q_a_, Q_b_ and Q_c_) and one R-SNARE, which contain a glutamine or an arginine in the 0-position, respectively (Fasshauer et al., 1998).

One of the earliest membrane fusion events in the secretory pathway is the anterograde transport between endoplasmic reticulum (ER) and Golgi. In this step, the Q_b_-SNARE GOSR2, also referred to as membrin or GS27, forms a complex with the Q_a_-SNARE syntaxin-5, the Q_c_-SNARE Bet1 and the R-SNARE Sec22b (Hay et al., 1997; Lowe et al., 1997). This SNARE complex mediates several fusion processes between the ER, the ER-Golgi intermediate compartment (ERGIC) and the Golgi (Hay et al., 1998). The importance of GOSR2 in these processes is supported by the observations that knockdown of GOSR2 leads to a significant decrease in transport from ER to Golgi and interferes with Golgi maintenance (Gordon et al., 2010; Lowe et al., 1997). The structural, kinetic and regulatory mechanisms of the complex formation are unknown, although it is likely that the R-SNARE Sec22b interacts with a preformed ternary complex of all three Q-SNAREs (Xu et al., 2000). It is also likely that this process is highly regulated: studies with yeast SNAREs *in vitro* have revealed that membrane fusion occurs only when Bet1 is located on a donor membrane and its SNARE partners are on an acceptor membrane (Parlati et al., 2000).

Mutations in GOSR2 are associated with progressive myoclonus epilepsies (PMEs), characterized by myoclonus, generalized tonic clonic seizures, and ataxia (Berkovic et al., 1986). A group of PME patients has been identified to possess a homozygous mutation of c.430G>T in the gene encoding for GOSR2 on chromosome 17, resulting in a p.Gly144Trp substitution in the protein. Glycine 144 is localized in the conserved SNARE domain of the protein. This homozygous mutation was first detected in a PME patient with severe motor disturbance with no described development of dementia. However, upon autopsy, a slightly reduced weight of the brain was measured and minor loss of Purkinje cells and gliosis in the cerebellar vermis were detected (Corbett et al., 2011). With time, 11 more patients were found to possess the p.Gly144Trp variant of GOSR2. The syndrome was called ‘North Sea PME’ given the fact that all patients originated from countries surrounding the North Sea (Boisse Lomax et al., 2013). These patients shared a similar phenotype, with an onset of ataxia of about 2 years, onset of myoclonic seizures of about 6.5 years and scoliosis by adolescence. The patients also do not display significant intellectual disability or cognitive dysfunction until late in disease progression.

**Abbreviations and nomenclature**

PMEs: Progressive myoclonus epilepsies
GOSR2: Golgi SNAP receptor complex member 2

Recently, a case of PME was reported in a female patient with compound heterozygous mutations in the gene encoding for GOSR2 (Praschberger et al., 2015). This patient carried the already described p.Gly144Trp mutation on one allele, whereas the other allele carried a previously unseen in-frame deletion of three base pairs, c.491_493delAGA. This deletion results in loss of a lysine (p.Lys164del) located within the SNARE domain of GOSR2. The respective patient was 61 years old and displayed a rather mild disease course with only mild cognitive dysfunction compared to patients homozygous for the p.Gly144Trp mutation. These observations suggest that the deletion of the lysine residue at position 164 has less severe functional consequences than the p.Gly144Trp mutation. Therefore, in this study we aimed to understand the functional effect of the two PME-linked mutations in GOSR2 and their influence on the stability and formation of the SNARE complex necessary for ER-to-Golgi transport.

## RESULTS

### The deletion of arginine 196 in Bos1 leads to a partial loss of function in yeast

The more mild disease course of the patient that is compound heterozygous for the p.Gly144Trp and the p.Lys164del mutations in GOSR2, and the lack of functional data on the p.Lys164del mutation, prompted us to functionally characterize these mutations in more detail. Both mutations reside in the SNARE domain of GOSR2 (Fig. 1) and therefore possibly affect SNARE complex assembly and/or function. The 16 layers of the SNARE domain of GOSR2 display remarkably evolutionary conservation in the animal kingdom and in the orthologous protein in fungi, called Bos1. Sequence alignment of human GOSR2 and Bos1 from *Saccharomyces cerevisiae* indicates conservation of the p.Gly144/p.Gly176 and similarity of the p.Lys164/p.Arg196 amino acids (Fig. 1). The p.Gly144/p.Gly176 residues are located within the −3 layer in the SNARE domain of both GOSR2 and Bos1, whereas the p.Lys164/p.Arg196 residues reside between layer +2 and +3 (Fig. 1).

**Fig. 2.**
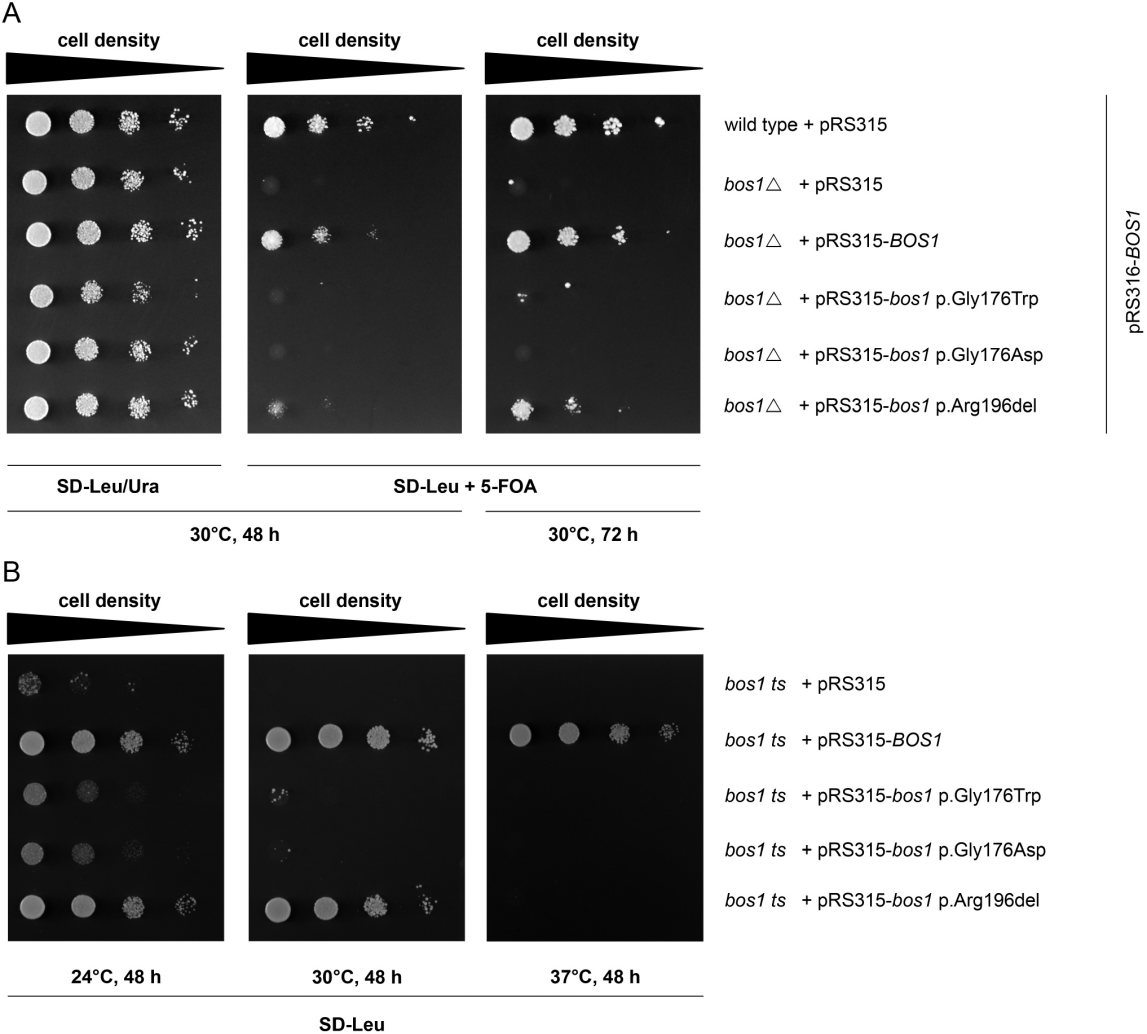
**The effect of Bos1 mutations on yeast growth.** (A) Bos1 p.Gly176Trp, p.Gly176Asp and p.Arg196del show growth impairment compared to Bos1 wild type. The yeast strain BY4742 *bos1*Δ containing pRS316-*BOS1* was generated as previously described (Corbett et al., 2011) and transformed with pRS315 plasmids expressing Bos1 wild type or Bos1 mutants. URA3, which is only present on the pRS316 plasmid, converts 5-fluoroorotic acid (5-FOA) to cytotoxic 5-fluorouracil. Plating yeast cells on media containing 5-FOA allows for negative selection regarding RS316. Only cells that express a functional Bos1 protein and lack pRS316 can thrive on 5-FOA-containing media. Indicated strains were spotted on SD-Leu/Ura and 5-FOA plates with serial dilutions (1:10) and incubated at 30°C for 48 h. Cells transfected with pRS315-*bos1*-p.Arg196del showed impaired growth compared to pRS315-*BOS1* wild-type strains; however, pRS315-*bos1*-p.Gly176Trp and -p.Gly176Asp led to severe impairment, indicating a functional impairment of the Bos1 p.Arg196del mutant and even stronger impairment of the Bos1 p.Gly176Trp and p.Gly176Asp variants. (B) Temperature-sensitive (ts) strain of *S. cerevisiae* was transformed with different variants of Bos1. At room temperature, growth was detected for all variants, including the negative control. At 24°C, reduced growth was detected for Bos1 p.Gly176Trp and p.Gly176Asp; only a few remaining colonies were detected for the negative control. At 30°C, only the wild type and the p.Arg196del enabled the yeast to grow. Finally, at 37°C, also yeasts transformed with the p.Arg196del variant lacked the ability to grow completely.

To investigate the functionality of the p.Gly176Trp and the p.Arg196del mutations in Bos1, we used a negative selection assay as described previously (Corbett et al., 2011). Because the *bos1*Δ strain of *S. cerevisiae* is not viable, a BY4742 wild-type strain was transformed with pRS316-*BOS1* followed by *BOS1* deletion to generate the endogenous *bos1*Δ knockout. Transformation with pRS315 plasmids encoding for Bos1 wild-type or Bos1 mutants followed by plating on media containing 5-fluoroorotic acid (5-FOA) allows for selection against pRS316 (Fig. 2A). Expression of the Bos1 p.Gly176Trp mutant completely perturbed growth after 48 h and conferred only a little growth after 72 h when compared to wild-type Bos1, indicating a very severe but not complete loss of function, as was reported previously (Corbett et al., 2011). Next, we substituted the glycine residue at position 176 for a hydrophilic aspartate to test the importance of the presence of a hydrophobic amino acid in the −3 layer. Yeasts expressing the p.Gly176Asp mutation also displayed complete perturbation of growth, suggesting that either a bulky hydrophobic or a hydrophilic amino acid at this position severely affects Bos1 function (Fig. 2A). By contrast, examination of the deletion of arginine 196 revealed only a slight growth perturbation after 48 and 72 h, indicating a less severe loss of function for this mutation.

To analyze the severity of these mutations on yeast growth more precisely and to avoid toxic side effects of 5-FOA, we transformed a temperature-sensitive yeast strain with the different Bos1 variants (Andag et al., 2001). At 24°C, the negative control showed reduced growth due to temperature sensitivity, whereas expression of wild-type Bos1 was able to rescue growth (Fig. 2B). Examination of yeasts expressing the p.Gly176Trp or the p.Gly176Asp mutation revealed significantly less growth than the strain carrying wild-type Bos1. The p.Arg196 deletion conferred comparable growth to the yeast strain transformed with wild-type Bos1 at 24°C. Increasing the temperature to 30°C resulted in an almost complete defect in growth of yeasts expressing the −3 layer mutations p.Gly176Trp or p.Gly176Asp, whereas only a slight decrease of growth for yeast carrying the p.Arg196 deletion was detected relative to wild type (Fig. 2B). Furthermore, at 37°C, yeasts expressing the p.Arg196del mutation displayed defective growth, whereas yeasts transformed with wild-type Bos1 were still able to thrive. Next, we aimed to analyze whether p.Lys164 and p.Arg196, in GOSR2 and Bos1, respectively, behaved similarly. Additionally, we wanted to examine whether an amino acid with a smaller side chain (p.Gly176Ala; rather than a large Trp or Asp) in the −3 layer can be tolerated. Therefore, we expressed Bos1 p.Arg196Lys and p.Gly176Ala variants in the temperature-sensitive Bos1 strain and detected similar growth for both variants and wild-type Bos1 (Fig. S1), strongly suggesting that these amino acid substitutions are functionally redundant at these positions.

### Altered assemblies of SNARE complexes containing the Bos1 p.Gly176Trp or p.Arg196del mutations

**Fig. 3.**
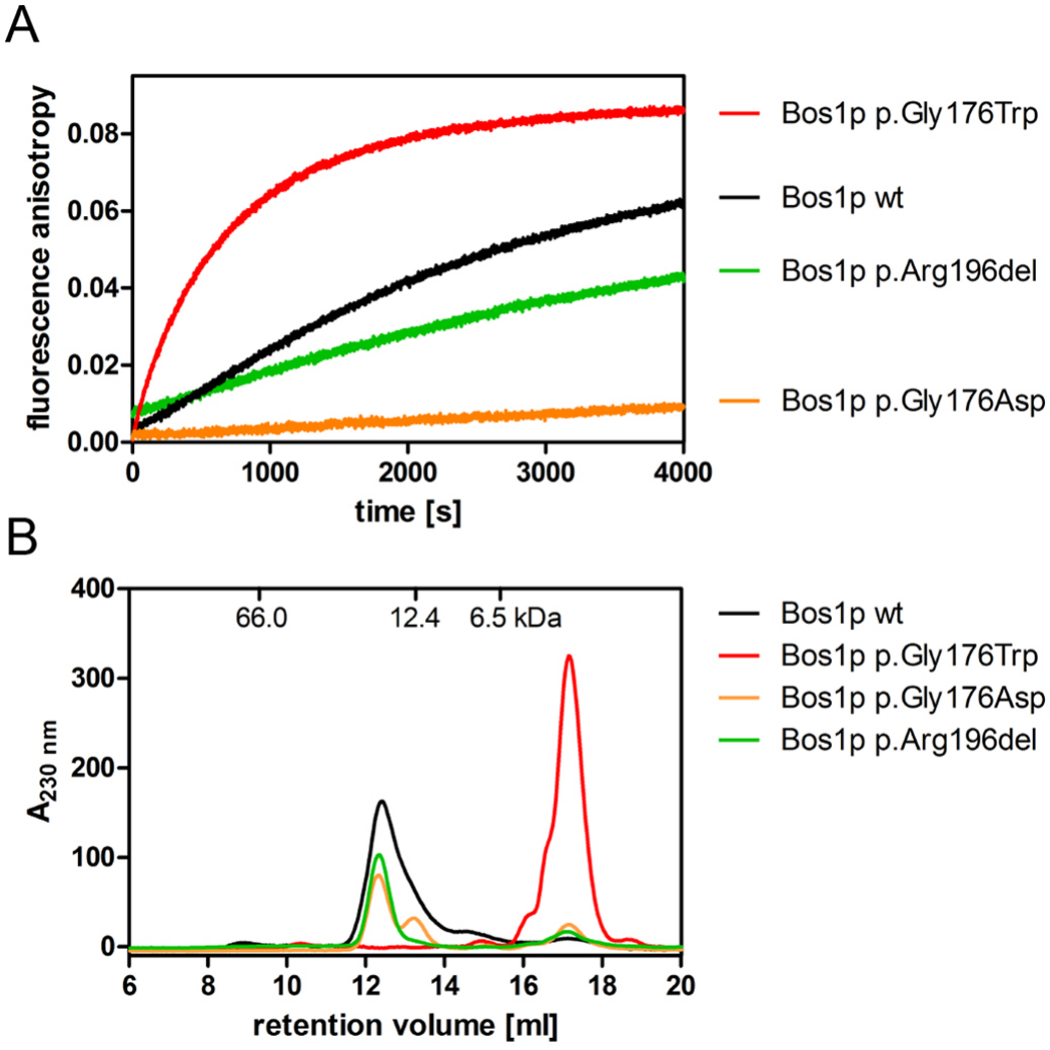
**ER-to-Golgi SNARE complex formation with Bos1 p.Arg196del.** (A) SNARE complex formation was measured by the increase of fluorescence anisotropy as previously described (Demircioglu et al., 2014). For assembly, 500 nM OG-labeled Sec22 126-186^C131OG^ were mixed with 2.7 µM Sed5 211-320, Bet1 1-118 and Bos1 151-221 or its mutants. A slower increase in fluorescence anisotropy indicates a reduced capability of Bos1 p.Arg196del to form the ER-to-Golgi SNARE complex. Bos1 p.Gly176Asp shows an even stronger effect. However, complex formation of Bos1 p.Gly176Trp seems to be accelerated. (B) Size exclusion chromatography (SEC) was performed for the individual SNARE domains of Bos1 variants. The Bos1 variants wild-type (wt), p.Gly176Asp and p.Arg196del seemed to be present in a higher oligomeric state than Bos1 p.Gly176Trp.

The reduced, but not complete, loss of function of the mutant Bos1 p.Gly176Trp and p.Arg196del in yeast growth experiments suggests that SNARE complex function is impaired but likely not completely abolished. Because SNARE complex assembly is a prerequisite for SNARE-mediated fusion of membranes, we analyzed the assembly properties of the ER-to-Golgi SNARE complex formed by Bos1, Bet1, Sed5 and Sec22 using fluorescence anisotropy measurements and size exclusion chromatography (SEC). We then compared the assembly behavior of the complex carrying wild-type Bos1 to complexes containing the Bos1 p.Gly176Trp and p.Arg196del mutations. SEC experiments revealed that both the wild-type Bos1 or the Bos1 SNARE domains carrying either of the two PME-associated mutations, p.Gly176Trp or p.Arg196del, or the designed p.Gly176Asp mutation, were able to assemble into complexes (Fig. S2). To examine the kinetics of Bos1 p.Gly176Trp and p.Arg196del assembling with Sec22, Sed5 and Bet1, we used fluorescence anisotropy measurements. This analysis allows the examination of SNARE complex formation *in vitro* using the isolated SNARE domains only, as described previously (Demircioglu et al., 2014). As expected, an increase of anisotropy of the labeled SNARE domain of Sec22 upon mixing with the SNARE domains of the respective complex partners (Bet1, Sed5 and Bos1) was detected, demonstrating the kinetics of SNARE complex assembly (Fig. 3A). Examination of the kinetics of complex formation for the p.Arg196del mutant revealed a slower rate of assembly. Interestingly, the p.Gly176Trp mutation induced a strongly augmented rate of assembly compared with wild-type Bos1. Taken together, these data indicate that both mutants are able to form SNARE complexes (Fig. 3A). To further dissect the faster SNARE complex formation kinetics of Bos1 carrying the p.Gly176Trp mutation, we also analyzed the p.Gly176Asp mutation because this substitution resulted in a similar attenuation of growth in yeast. In contrast to the p.Gly176Trp variant, complex formation of the Bos1 p.Gly176Asp variant was severely reduced when measured by the fluorescence anisotropy assay (Fig. 3A). These data suggest that the introduction of a hydrophilic amino acid at the p.Gly176 position has a profound effect on the speed of SNARE complex assembly.

Next, we wanted to understand the faster kinetics of SNARE complex assembly for the Bos1 p.Gly176Trp mutant. Therefore, we analyzed the effects of Bos1 mutations on homo-oligomer assembly using SEC of the purified SNARE domains. Analysis of the p.Gly176Asp and p.Arg196del mutations revealed a significant propensity to form oligomers, which was similar to wild-type Bos1. In contrast the retention volume of Bos1, that of p.Gly176Trp was largely increased (Fig. 3B), strongly suggesting that the oligomerization capacity of p.Gly176Trp was significantly reduced. These data suggest that the increased rate of SNARE complex formation may be a result of the reduced propensity of the p.Gly176Trp mutation to form homo-oligomers, thereby increasing the number of monomeric Bos1 available for assembly.

### *In silico* simulation of PME mutations in GOSR2/Bos1 reveal disturbances in the SNARE complex

**Fig. 4.**
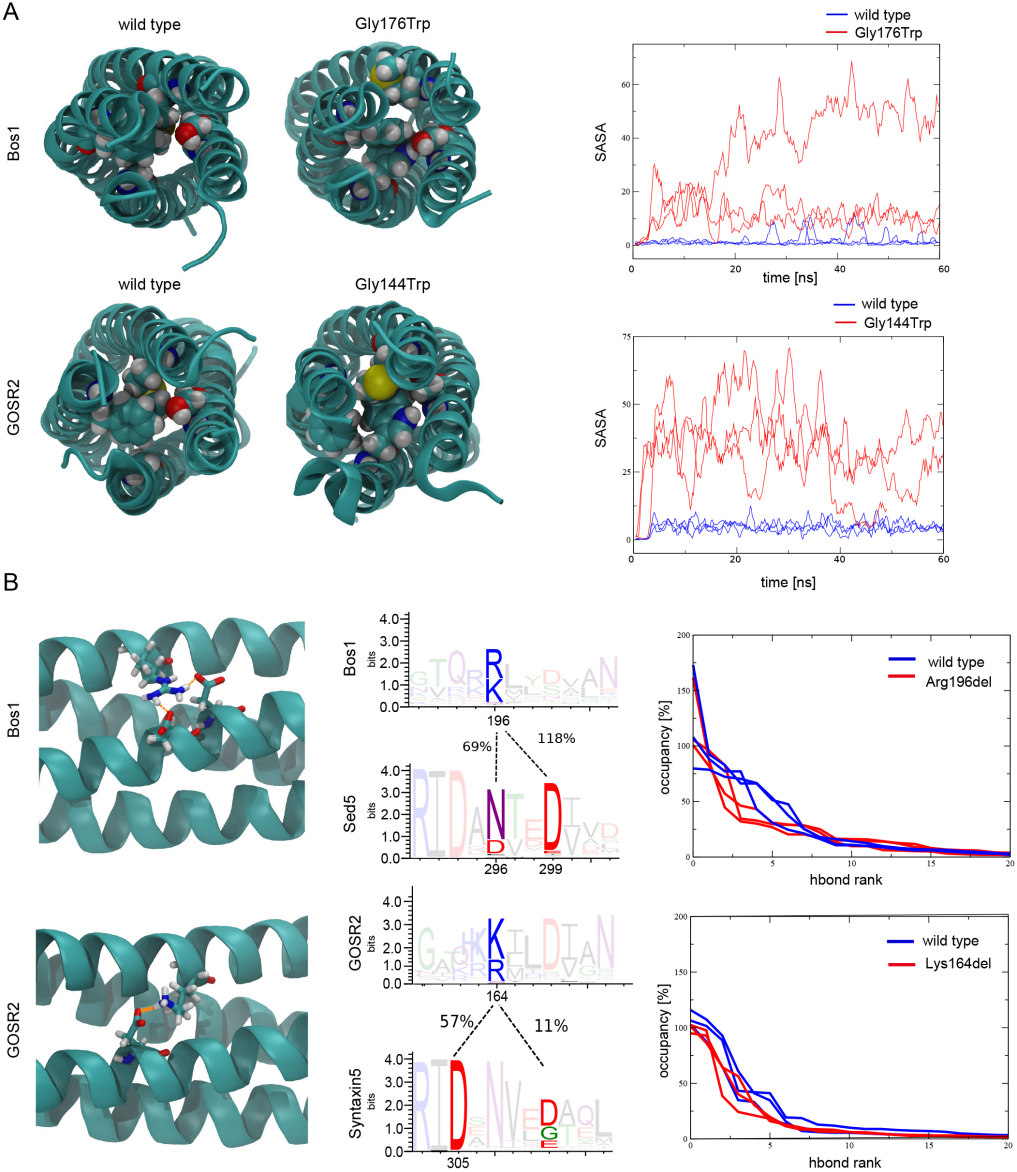
***In silico* simulation of the SNARE complexes bearing the two PME mutations.** Molecular dynamics (MD) simulations suggest that the impact on the SNARE complex of component proteins bearing mutated residues varies. (A) MD simulations of models of the quaternary yeast and human ER-to-Golgi SNARE complexes revealed that the Gly144Trp mutation of GOSR2 as well as the orthologous Gly176Trp mutation of Bos1 interfered with the integrity of the hydrophobic core of the helix bundle in the region surrounding layer −3, visible by an increase of the solvent-accessible surface area (SASA). Plotted are independent MD replicas. (B) The deletion of Lys164 in GOSR2 or Arg196 in Bos1 led to a slight impairment of the hydrogen bond network between Q_a_- and Q_b_-helices. On the right, relative hydrogen-bond occupancy for different hydrogen bonds between the neighboring Q_a_- and Q_b_-helices is reported for the different models. The different hydrogen bonds are sorted based on their occupancy (hbond rank).

Our results indicate that Bos1 p.Gly176Trp and p.Arg196del are able to assemble into SNARE complexes with Sed5, Sec22 and Bet1, whereas the p.Gly176Asp mutation displayed severely attenuated complex formation. Because there is no crystal structure available for this particular SNARE complex, we modeled the quaternary yeast and human ER-to-Golgi SNARE complex using available SNARE complex structures as template. These homology models were then explored by MD simulations (Fig. 4). Our results reveal that the wild-type model of the assembled quaternary complexes remained stable within a 10^2^ ns timescale, as shown by root mean square deviation (RMSD) profiles (Fig. S3A). Comparable results were obtained when we used existing X-ray structures of SNARE complexes (data not shown), corroborating the robustness of our SNARE complex models. We next simulated with MD complexes carrying the Bos1 p.Gly176Trp or p.Arg196del mutations and the human orthologs, which previous results have shown to be capable of complex formation (Fig. S2). Both PME mutations behaved similar to the wild-type protein, with both showing similarly stable RMSD values, suggesting that the mutated SNARE complexes are stable in the explored timescale (Fig. S3A). Next, we analyzed secondary-structure alterations during simulations. As depicted in Fig. S3B, the SNARE complex bearing the Bos1 p.Gly176Trp mutation and the human ortholog showed no strong alterations in secondary structure. However, we observed small changes in the N-terminal region, bearing the GOSR2 p.Gly144Trp mutation. In contrast, the complexes carrying the Bos1 p.Arg196del or GOSR2 p.Lys164del mutation exhibited a local loss of helical structure, likely due to the lack of helical periodicity (Fig. S3B).

Although complexes containing mutated Bos1 or GOSR2 remained stable during prolonged MD simulations, the structures appeared to exhibit local structural disturbances. We investigated the regions immediately surrounding the mutations in more detail. We noted that the −3 layer, containing the p.Gly176Trp exchange, is highly asymmetric. In addition to the glycine from Bos1, the −3 layer is composed of a methionine from Sec22, a phenylalanine from Sed5 and a serine from Bet1. As expected, the substitution of the small and highly conserved glycine to a bulky tryptophan makes it difficult to pack all four side chains of this layer into the core of the complex. Our MD simulations reveal that adjacent residues opposing Trp176 in the hydrophobic layer avoided steric clashes and repeatedly evaded the hydrophobic core of −3 layer, getting more solvent-accessible (Fig. 4A). Similar results were obtained for the mutation of the −3 layer of the orthologous human complex, which has the same amino acid composition (Fig. 4A). This observation is consistent with our yeast growth experiments where the substitution of glycine for another small residue, an alanine, did not lead to growth impairment (Fig. S1), whereas substitution to a large tryptophan led to a clear growth defect (Fig. 2).

The deletion of the arginine residue at position 196 of Bos1 or the lysine residue at position 164 of GOSR2 had a different effect on the assembled SNARE complex. Our MD simulations showed that the deletion was surprisingly well tolerated in the assembled four-helix bundle structure, although it produces a significant discontinuity of the coiled-coil heptad repeat and tighter local winding (Fig. 4B). In contrast to the Gly176Trp mutation, we did not observe a significant change in the solvent-accessible surface area (SASA) of the adjacent residues contributing to the hydrophobic core during simulations (Fig. S3C). It is possible that the p.Arg196del mutation does not affect the hydrophobic core of the complex, but instead changes the arrangement of residues that shield the interior of the bundle and alters hydrogen bonding. In the wild-type complex, the deleted arginine residue at position 196 is involved in stable hydrogen bond formation with an aspartate residue at position 299 in Sed5 (Fig. 4B). Comparison of the distribution of hydrogen bonds between Bos1 and Sed5 showed that the hydrogen bond network was affected by the deletion of the p.Arg196 residue, likely as a result of reduced numbers of contacts between the two neighboring SNARE helices (Fig. 4B).

In summary, we have shown, by using the orthologous yeast ER-to-Golgi SNARE complex, that the pathogenic mutations p.Gly144Trp and p.Lys164del in the Q_b_ SNARE GOSR2 do not interfere with general SNARE complex stability. However, the p.Gly144Trp mutation in the −3 layer appears to disturb the stability and solvent accessibility of the hydrophobic core of the SNARE complex, whereas the p.Lys164del mutation perturbs hydrogen bonding between GOSR2 and syntaxin-5. Finally, yeast growth experiments demonstrate that the p.Gly144Trp mutation causes a more severe phenotype than the p.Lys164del mutation, which corresponds well with the progression of the disease in PME patients. Thus, our data suggest that a combination of *in silico* and yeast experiments can describe at a molecular level the assembly and stability of variant SNARE complexes.

## DISCUSSION

GOSR2 is a Q_b_-SNARE protein involved in ER-to-Golgi trafficking, and is associated with PME. In the present study, we investigated the functionality of the PME-associated GOSR2 mutations p.Gly144Trp and p.Lys164del using yeast orthologs.

Yeasts carrying the orthologous p.Arg196del mutation of Bos1 showed growth defects at elevated temperatures typical for a temperature-sensitive strain. This finding suggests that the function of mutated Bos1 and the orthologous mutation in GOSR2 p.Lys164del is impaired but not lost. In contrast, the Bos1 p.Gly176Trp (GOSR2 p.Gly144Trp) mutation led to a more significant impairment of function in yeast as indicated by severely impaired growth even at lower temperatures. These results are consistent with the milder disease phenotype observed in the patient carrying the compound heterozygous p.Lys164del and p.Gly144Trp mutations, compared to patients homozygous for the p.Gly144Trp mutation (Boisse Lomax et al., 2013; Praschberger et al., 2015).

Physical simulations of protein complexes indicate that the p.Gly144Trp mutation affects the stability of the hydrophobic core, which provides the driving force for SNARE complex formation. The substitution of the small glycine to a large tryptophan causes steric clashes that might interfere with assembly of the four-helix bundle, but might also destabilize the entire complex, thereby reducing its vesicle-fusion activity. Notably, the p.Gly144Trp mutation is in close proximity to a site important for v-SNARE binding (Pobbati et al., 2006; Wiederhold and Fasshauer, 2009). Highly decelerated complex formation of p.Gly176Asp but acceleration of the p.Gly176Trp variant is contrary to the expectations for impaired SNARE function. However, we found that the glycine-to-tryptophan substitution also changed the oligomeric state of Bos1 in our *in vitro* assembly experiments. Whereas the isolated SNARE domain of wild-type Bos1 was present as an oligomer, which needs to dissociate first for SNARE complex formation, Bos1 p.Gly176Trp was already present as a reactive monomer. It is unlikely, though, that oligomerization of the SNARE domain of Bos1 plays a role during SNARE complex formation *in vivo*.

According to our data, microdeletion of lysine 164 did not affect the hydrophobic core, but reduced the occupancy of particular hydrogen bonds between Q_a_ and Q_b_ helices. This microdeletion led to a slightly destabilized α-helix in the second half of the complex. This has a less significant impact on complex assembly because the N-terminal part contributes more to complex assembly (Pobbati et al., 2006). This is supported by fluorescence anisotropy measurements, which displayed only slightly slower complex assembly of Bos1 p.Arg196del with its SNARE partners Sed5, Bet1 and Sec22, compared to wild-type Bos1. The effect of the deletion was far less severe than observed for the p.Gly176Asp variant. Although the deletion does not impinge on the stability of the hydrophobic core, it might still affect the membrane-fusion activity of the assembled complex, because of the tighter local winding of the Q_b_-helix close to the transmembrane region.

In summary, our study provides an analysis of PME-associated GOSR2 mutations *in silico* and *in vitro*. We show that the milder course of disease in a compound heterozygous PME patient for GOSR2 p.Gly144Trp and p.Lys164del, when compared to patients homozygous for GOSR2 p.Gly144Trp, is due to less severe impairment of SNARE function by the p.Lys164del mutations. We also investigated SNARE function at the molecular level and showed that p.Gly144Trp interfered with the SNARE hydrophobic core, whereas the p.Lys164del mutation perturbed hydrogen bond formation between GOSR2 and syntaxin-5. We propose that the observed SNARE complex malfunction due to both mutations could result in impaired fusion of ER- and ERGIC-derived vesicles with the cis-Golgi target membrane, leading to a perturbation of ER-to-Golgi trafficking. In neurons, the impairment of the early anterograde transport might lead to disorders such as epilepsy due to alterations in the regulated release of neurotransmitters, as well as the proper sorting of neurotransmitter receptors at chemical synapses, providing a possible link between mutations in GOSR2 and epilepsy (Giannandrea et al., 2010; Multani et al., 1994).

## MATERIALS AND METHODS

### Materials

If not stated otherwise, chemicals were obtained from Merck (Darmstadt, Germany), Roth (Karlsruhe, Germany), Thermo Fisher Scientific (Bremen, Germany) or Sigma-Aldrich (Steinheim, Germany).

### Yeast strains and negative selection with 5-FOA

Yeast strains and plasmids used in this study are described in Table S1. The BY4742 *bos1*Δ strain was generated by transformation of BY4742 with pRS316-*BOS1*. The resulting strain was transformed with *bos1* deletion cassettes produced by PCR to achieve the endogenous *bos1* deletion as previously described (Corbett et al., 2011). BY4742 *bos1*Δ strains containing pRS316-*BOS1* and pRS315 plasmids expressing Bos1, Bos1 p.Gly176Trp, Bos1 p.Gly176Asp or Bos1 p.Arg196del were grown for 12 h in standard minimal medium with appropriate supplements (SD-Leu/Ura). Equal ODs with serial dilutions (1:10) were plated on SD-Leu/Ura or SD-Leu containing 5-FOA and incubated at 30°C for 48 h. Plating of the indicated yeast strains on media containing 5-FOA allowed for negative selection regarding pRS316.

### Yeast strains and survival assay of temperature-sensitive yeast strain

Yeast strains and plasmids used in this study are described in Table S1. The temperature-sensitive (ts) yeast strain *bos1 ts* S32G-8A (Andag et al., 2001) cannot thrive at 30°C or higher temperatures, unless a functional copy of *bos1* is transformed and expressed. The *bos1* ts strains containing pRS315 plasmids expressing Bos1, Bos1 p.Gly176Trp, Bos1 p.Gly176Asp or Bos1 p.Arg196del was grown for 12 h in standard minimal medium with appropriate supplements (SD-Leu). Equal ODs with serial dilutions (1:10) were plated on SD-Leu and incubated at 24, 30 or 37°C for 48 h or room temperature for 72 h. Only yeast strains expressing a functional copy of Bos1 can rescue the ts phenotype of the *bos1* ts strain at 30°C and 37°C.

### Protein constructs and purification

Plasmids used in this study are listed in Table S2. Unlike Bet1, only the SNARE domains of Sed5, Sec22 and Bos1 were expressed. Single-cysteine variants used in this study were designed as previously described (Demircioglu et al., 2014). Recombinant proteins were expressed in *Escherichia coli* strain BL21 (DE3) and purified by Ni^2+^-NTA chromatography followed by ion exchange chromatography on an Äkta system (GE Healthcare, Solingen, Germany). Depending on the pI of each protein, MonoQ or MonoS was used as ion exchanger. Protein elution was performed by using a linear gradient of NaCl in 20 mM Tris, pH 7.4 buffer containing 1 mM EDTA and additionally 1 mM DTT for proteins carrying cysteine residues. Hexa-His tags were removed before ion exchange chromatography via thrombin cleavage. Protein concentrations were determined by absorption at 280 nm or using Bradford assay.

### Fluorescence anisotropy

Fluorescence measurements were performed in a spectrofluorometer equipped with a second emission channel in T-configuration (QuantaMaster 40, PTI, Birmingham, NJ). Sec22 126-186^D131C^ was labeled with Oregon Green (OG) 488 iodoacetamide according to the manufacturer's protocol and protein concentration determined via Bradford assay. Experiments were carried out in 1-cm quartz cuvettes (Hellma, Müllheim, Germany) in PBS buffer at 25°C. Measurement of fluorescence anisotropy, which increases upon complex formation due to local flexibility of the labeled residue, was carried out as previously described (Burkhardt et al., 2008).

### Size exclusion chromatography (SEC)

The oligomeric state of single proteins and quaternary SNARE complexes was analyzed by SEC on a Superdex 75 column in PBS buffer containing 200 mM NaCl. Quaternary SNARE complexes consisting of Sed5 (211-320), Bet1 (1-118), Sec22 (126-186) and one of the Bos1 (151-221) variants were assembled with equal amounts of purified components and incubated overnight in PBS buffer containing 200 mM NaCl. The molecular weight was calculated with a standard containing Dextran blue, BSA, ovalbumin, cytochrome *c* and aprotinin.

### Modeling and MD simulations of the SNARE complexes

A set of crystal structures of different SNARE complexes (PDB ID codes 2NPS, 2GL2, 1SFC, 3B5N, 4WY4) was used as templates for modeling (Welch et al., 2012). Models of human and yeast complexes were generated with Modeller v.9 (Sali and Blundell, 1993). Each complex was prepared for simulations using the Leap module of AmberTools (Schafmeister et al., 1995). Simulations were run with the NAMD engine (Phillips et al., 2005) using the AMBER99SBildn force field (Hornak et al., 2006) and TIP3P parameters for water (Jorgensen et al., 1983). Standard sodium and chloride parameters from the AMBER force field were used. A conservative cutoff of 12 Å (Piana et al., 2012) was set for nonbonded interactions with a switching function active between 10 and 12 Å. Electrostatics were treated through particle-mesh Ewald summations with a grid spacing of 1 Å. Each simulation box was minimized, equilibrated by Cα-restrained heating in ten steps of 30 K up to 300 K for a total of 1 ns, and further equilibrated by unrestrained heating. Subsequently, the production simulations were carried out at 300 K and 1 atm, controlled with a Nosé–Hoover Langevin piston.

Models of human and yeast complexes after unrestrained equilibration were used as templates to generate corresponding mutant complexes. After equilibration, all models were simulated for 90 ns. Because no significant RMSD changes were observed after the first 50 ns, additional replicas were only run for 60 ns. Each system was simulated in three replicas. Trajectories were analyzed with VMD software modules and Tcl scripts. For hydrogen bond contact measurements, a cutoff distance of 3.6 Å between heavy atoms and an angle cutoff of 30° were used.

## Supplementary Material

Supplementary information
