# Supplementary material for: Functional assays for the assessment of the pathogenicity of variants of GOSR2, an ER-to-Golgi SNARE involved in progressive myoclonus epilepsies
Source: Dis Model Mech. 2017 Dec 1;10(12):1391–8. doi: 10.1242/dmm.029132 (PMC5769602; doi:10.1242/dmm.029132)
Supplement: Supplementary information [file dmm-10-029132-s1.pdf]

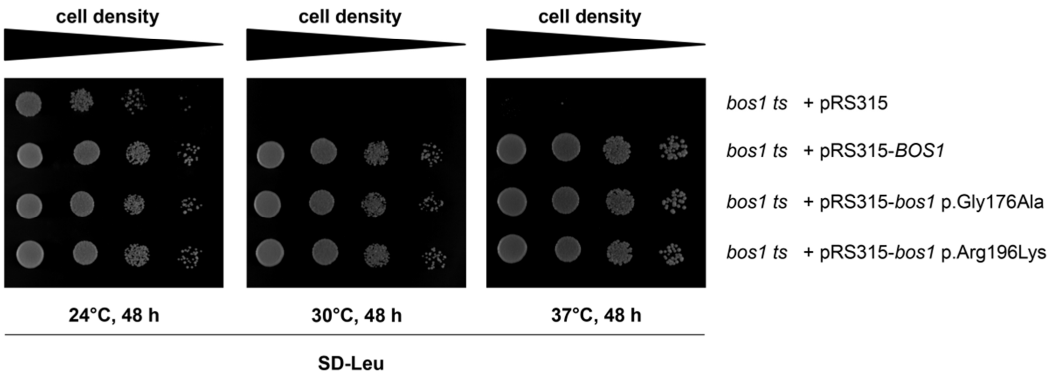

Fig. S1:

We used a temperature sensitive *bos1 ts* strain expressing Bos1 p.Arg196Lys and p.Gly176Ala variants for yeast growth experiments. We detected similar growth for both variants when compared to wild type Bos1, suggesting that these amino acid substitutions are functionally redundant at these positions.

Disease Models & Mechanisms • Supplementary information

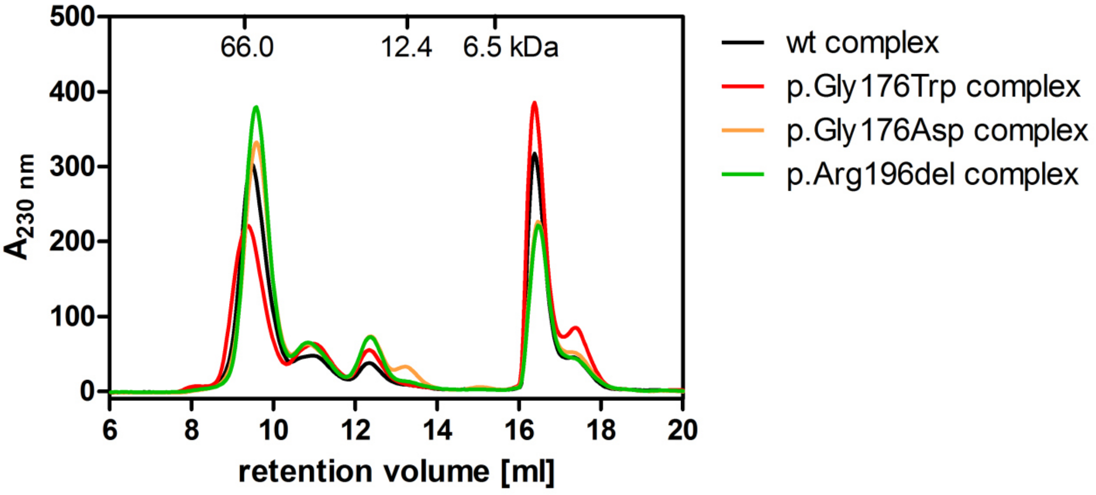

Fig. S2:

Size exclusion chromatography of the Bos1 variants wt, p.Gly176Trp, p.Gly176Asp and p.Arg196del after incubation with its complex partners Sec22, Bet1 and Sed5 revealed that all variants are capable of complex formation.

Disease Models & Mechanisms • Supplementary information

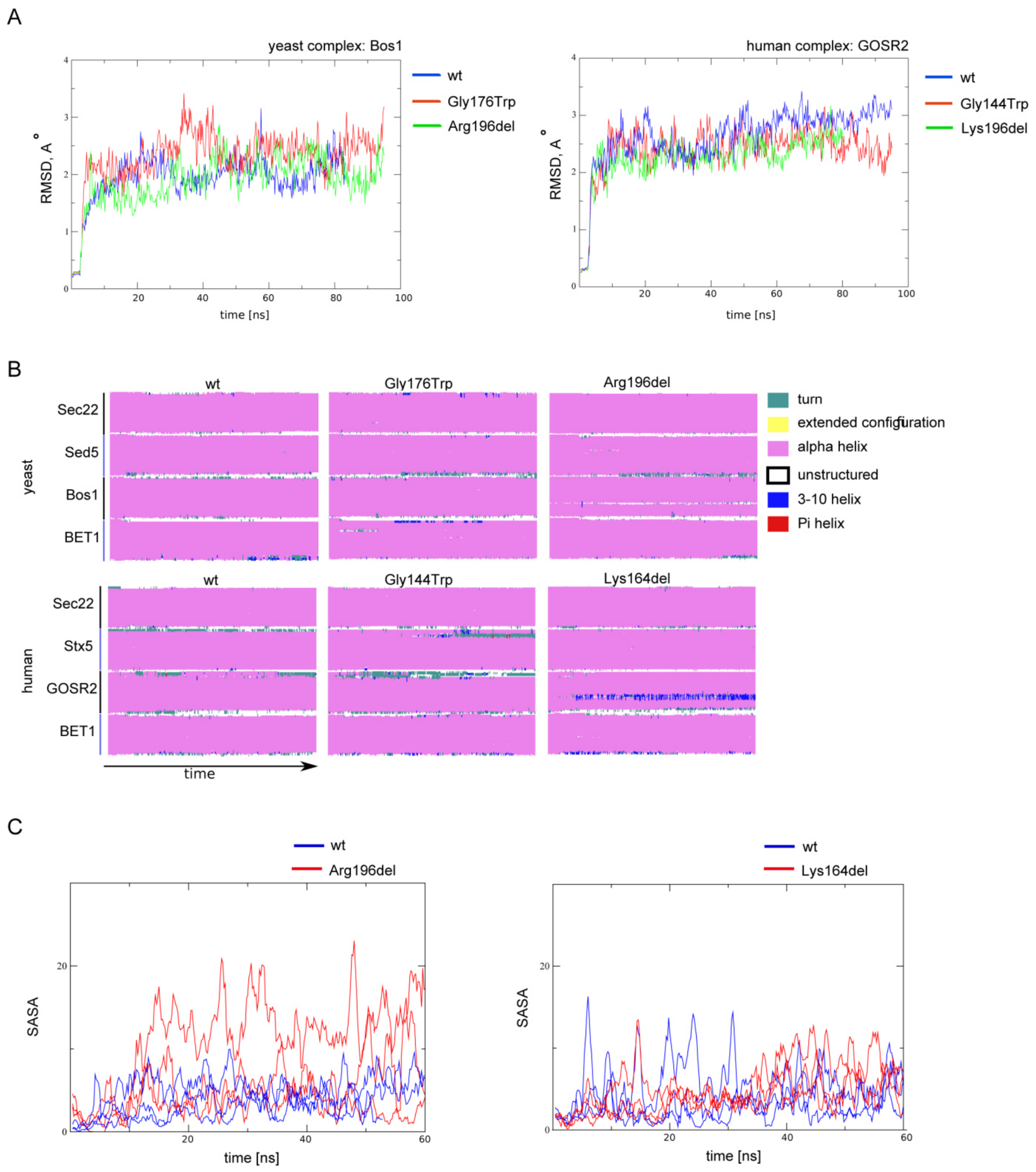

Fig. S3:

(A) Root mean square deviation (RMSD) measurement indicates that the assembled quaternary complexes of all Bos1 and GOSR2 variants with the complex partners are in principle stable. (B) GOSR2 p.Gly144Trp mutations showed no obvious alterations in secondary structure for the SNARE complex bearing the Bos1 p.Gly176Trp mutation and the human orthologue, even if the N-terminal part in the complex bearing the GOSR2 p.Gly144Trp mutation was slightly impaired. However, the complexes carrying the Bos1 p.Arg196del or GOSR2 p.Lys164del mutation exhibited a local loss of helical structure, likely due to the lack of helical periodicity. (C) No significant change in the solvent accessible surface area (SASA) during simulations was observed for the SNARE complexes bearing the Bos1 p.Arg196del or the GOSR2 p.Lys164del mutation.

Table S1: Organisms used in this work

Organism	Strain	Genotype	Source
Saccharomyces cerevisiae	BY4742	MATα *his3Δ1 leu2Δ0 ura3Δ0 lys2Δ*	(Winzeler et al., 1999)
Saccharomyces cerevisiae	BY4742 *bos1Δ*	*MATα his3Δ1 leu2Δ0 ura3Δ0 lys2Δ0 bos1Δ::KanMX6* pRS316-sc*BOS1*	(Corbett et al., 2011)
Saccharomyces cerevisiae	S32G-8A *bos1 ts*	*MATα, ura3, leu2, his3, sec32-1/bos1*	(Andag et al., 2001)
Escherichia coli	BL21 (DE3)	*E. coli* B F-*dcm ompT hsdS* (rB-mB-) Gal λ (DE3)	Novagen® (Merck, Darmstadt, Germany)

Table S2: Plasmids used in this work

Plasmid	Description	Source
pRS315	*CEN6, LEU2* based yeast-*E. coli* shuttle vector	(Sikorski and Hieter, 1989)
pRS316	*CEN6, URA3* based yeast-*E. coli* shuttle vector	(Sikorski and Hieter, 1989)
pRS315-*BOS1*	pRS315 shuttle vector expressing Bos1	(Corbett et al., 2011)
pRS315-*bos1*-p.Arg196del	pRS315 shuttle vector expressing Bos1 p.Arg196del	Produced within this work
pRS315-*bos1*-p.Gly176Trp	pRS315 shuttle vector expressing Bos1 p.Gly176Trp	(Corbett et al., 2011)
pRS315-*bos1*-p.Gly176Asp	pRS315 shuttle vector expressing Bos1 p.Gly176Asp	Produced within this work
pRS315-*bos1*-p.Gly176Ala	pRS315 shuttle vector expressing Bos1 p.Gly176Ala	Produced within this work
pRS315-*bos1*-p.Arg196Lys	pRS315 shuttle vector expressing Bos1 p.Gly176Ala	Produced within this work
pET28a-*sed5* (211-320)	bacterial expression vector for recombinant protein expression of N-terminal (thrombin-cleaveable) His6-tagged Sed5 (aa 211-320)	(Demircioglu et al., 2014)
pET28a-*bet1*-p.G85C (1-118)	bacterial expression vector for recombinant protein expression of N-terminal (thrombin-cleaveable) His6-tagged Bet1 p.Gly85Cys (aa 1-118)	(Demircioglu et al., 2014)
pET28a-*sec22*–p.Asp131Cys (126-186)	bacterial expression vector for recombinant protein expression of N-terminal (thrombin-cleaveable) His6-tagged Sec22 p.Asp131Cys (aa 1-118)	(Demircioglu et al., 2014)
pET28a-*bos1* (151-221)	bacterial expression vector for recombinant protein expression of N-terminal (thrombin-cleaveable) His6-tagged Bos1 (aa 151-221)	Produced within this work
pET28a-*bos1*-p.Arg196del (151-221)	bacterial expression vector for recombinant protein expression of N-terminal (thrombin-cleaveable) His6-tagged Bos1 p.Arg196del (aa 151-221)	Produced within this work
pET28a-*bos1*-p.Gly176Trp (151-221)	bacterial expression vector for recombinant protein expression of N-terminal (thrombin-cleaveable) His6-tagged Bos1 p.Gly176Trp (aa 151-221)	Produced within this work

Disease Models & Mechanisms • Supplementary information

pET28a-*bos1*-p.Gly176Asp (151-221)	bacterial expression vector for recombinant protein expression of N-terminal (thrombin-cleaveable) His6-tagged Bos1 p.Gly176Asp (aa 151-221)	Produced within this work

Andag, U., Neumann, T. and Schmitt, H. D. (2001). The coatomer-interacting protein Dsl1p is required for Golgi-to-endoplasmic reticulum retrieval in yeast. *J. Biol. Chem.,* **276,** 39150-60.

Corbett, M. A., Schwake, M., Bahlo, M., Dibbens, L. M., Lin, M., Gandolfo, L. C., Vears, D. F., O'sullivan, J. D., Robertson, T., Bayly, M. A., et al. (2011). A mutation in the Golgi Qb-SNARE gene GOSR2 causes progressive myoclonus epilepsy with early ataxia. *American journal of human genetics,* **88,** 657-63.

Demircioglu, F. E., Burkhardt, P. and Fasshauer, D. (2014). The SM protein Sly1 accelerates assembly of the ER-Golgi SNARE complex. *Proceedings of the National Academy of Sciences of the United States of America,* **111,** 13828-33.

Sikorski, R. S. and Hieter, P. (1989). A system of shuttle vectors and yeast host strains designed for efficient manipulation of DNA in Saccharomyces cerevisiae. *Genetics,* **122,** 19-27.

Winzeler, E. A., Shoemaker, D. D., Astromoff, A., Liang, H., Anderson, K., Andre, B., Bangham, R., Benito, R., Boeke, J. D., Bussey, H., et al. (1999). Functional characterization of the S. cerevisiae genome by gene deletion and parallel analysis. *Science (New York, N.Y.),* **285,** 901-6.
